# Independent modulations of the transmission amplitudes and phases by using Huygens metasurfaces

**DOI:** 10.1038/srep25639

**Published:** 2016-05-20

**Authors:** Xiang Wan, Sheng Li Jia, Tie Jun Cui, Yong Jiu Zhao

**Affiliations:** 1 State Key Laboratory of Millimeter Waves, School of Information of Science and Engineering, Southeast University, Nanjing 210096, China; 2 Synergetic Innovation Center of Wireless Communication Technology, Southeast University, Nanjing, 210096, China; 3 Key Laboratory of Radar Imaging and Microwave Photonics, Ministry of Education, Nanjing University of Aeronautics and Astronautics, Nanjing 210016, China; 4 Cooperative Innovation Center of Terahertz Science, Chengdu 611731, China

## Abstract

We propose ultrathin Huygens metasurfaces to control transmission amplitudes and phases of electromagnetic waves independently, in which each unit cell is comprised of an electric dipole and a magnetic dipole. By altering the electric and magnetic responses of unit cells, arbitrary complex transmission coefficients with modulus values smaller than 0.85 are obtained. Two Huygens metasurfaces capable of controlling the diffraction orders are designed and fabricated by modulating the distributions of the complex transmission coefficients. More complicated functions such as holographic imaging can also be accomplished by using the proposed Huygens metasurfaces.

As a kind of two-dimensional metamaterials, metasurfaces have attracted a lot of attentions in both engineering and science societies^1^,^2^,^3^,^4^,^5^,^6^,^7^,^8^,^9^,^10^,^11^. The geometrical features of metasurfaces alleviate the difficulties in engineering realizations, and bring new concepts and methods, such as the holography^12^,^13^,^14^,^15^ and generalized Snell’s law^8^,^16^,^17^ in designing functional devices^2^,^8^,^11^. Independent controls of amplitudes and phases are important requirements in practical applications such as beam shaping and synthesizes, which have been realized using different metasurfaces^18^,^19^,^20^,^21^,^22^,^23^,^24^,^25^,^26^, most of which, however, only manipulate the electric responses, thus leading to low efficiencies and limited abilities in fully controlling the electromagnetic waves. Huygens principle provides a method to design both of the electric and magnetic responses. Several functional metasurfaces have been proposed by using the Huygens principle^27^,^28^,^29^,^30^,^31^, and the ability of Huygens metasurfaces to control reflection amplitudes and phases independently has been confirmed in the optical region^32^, in which the maximum efficiency is 45%. Nevertheless, the transmission-type Huygens metasurfaces with capability to control both amplitudes and phases have not been reported.

Here, we propose high-efficiency and independent controls of the transmission amplitudes and phases by engineering both electric and magnetic responses of the proposed Huygens particles. Full-wave simulations show that arbitrarily complex transmission coefficients with modulus values less than 0.85 are available. Based on these efficient particles, two Huygens metasurfaces are designed and fabricated, which are capable of controlling the diffraction beams. The measurement results show very well agreements with numerical simulations. We remark that more complicated functions can be designed by modulating the distributions of transmission amplitudes and phases on the metasurfaces.

## Theories and analyses

**Figure 1:**
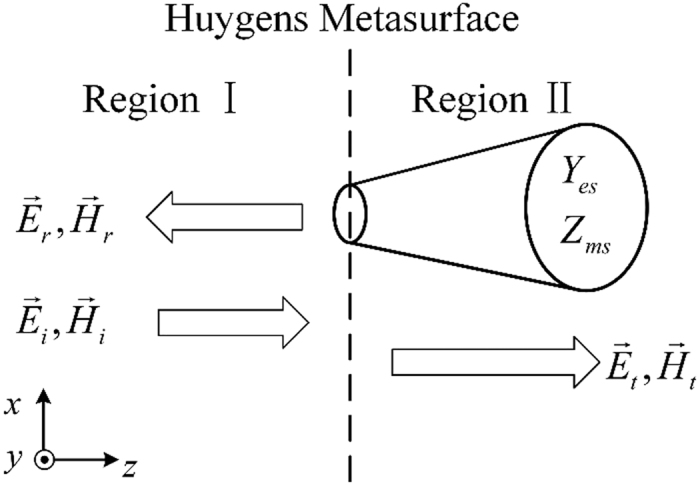
The schematic of a Huygens metasurface under normal incidence.

**Figure 2:**
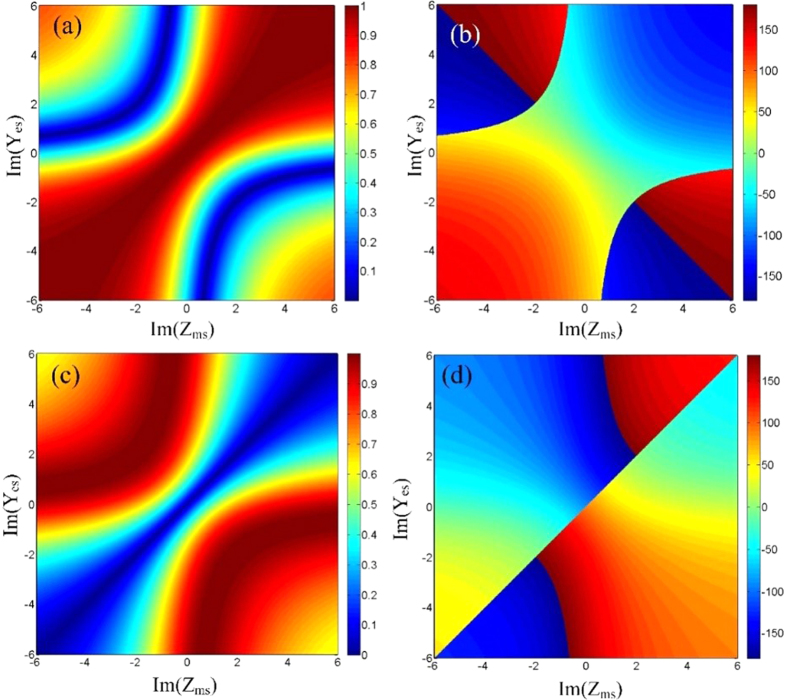
The amplitudes and phases of calculated transmission and reflection coefficients as functions of the surface electrics and magnetic impedances. (**a**) The transmission amplitudes. (**b**) The transmission phases. (**c**) The reflection amplitudes. (**d**) The reflection phases.

Figure 1 gives the schematic of a Huygens metasurface under the normal incidence. The Huygens metasurface can be characterized by the electric surface admittance 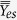 and magnetic surface impedance 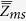. For a predetermined field distribution in Region Ι, arbitrary electromagnetic fields in Region ΙΙ can be produced by altering the electric surface admittance and magnetic surface impedance. According to the equivalence principle and the boundary condition, the electromagnetic fields in such two regions satisfy the following equations^24^,^33^.


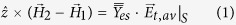



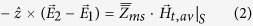


where 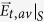 and 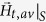 are the averaged tangential electric and magnetic fields on the surface, respectively^24^. 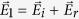, 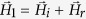 and 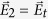, 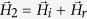 are the total fields in Regions Ι and ΙΙ, respectively. The transmission and reflection coefficients can be represented by the electric and magnetic surface impedances as^34^,^35^


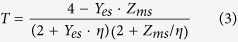



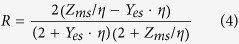


in where *η* is the wave impedance in free space. For simplicity, the electric and magnetic surface impedances are assumed to be isotropic (*Y*_*es*_ and *Z*_*ms*_) when the polarization of the incident wave is determined. Figure 2 gives the amplitudes and phases of the calculated transmission and reflection coefficients as functions of the surface electrics and magnetic impedances. The Huygens metasurface is assumed to be passive and lossless. It is observed that the phases tend to be unchanged when the amplitudes vary, and vice versa. This property indicates that the amplitudes and phases can be controlled independently.

**Figure 3:**
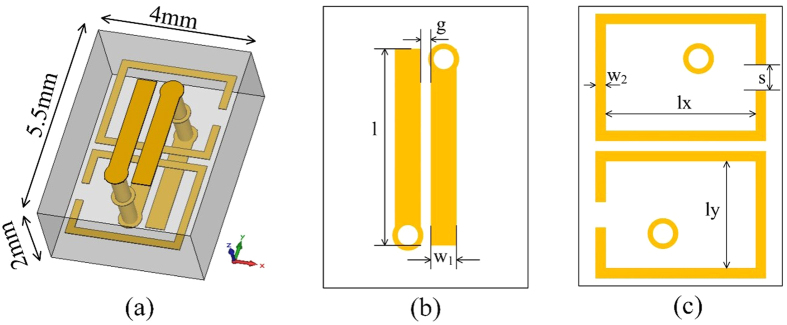
Detailed geometries of a Huygens particle. (**a**) Perspective view. (**b**) The geometries of the outer layer. (**c**) The geometries of the middle layer.

**Figure 4:**
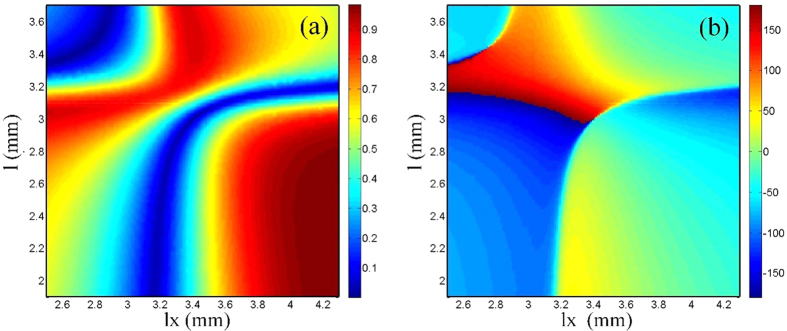
Parametric analyses when change the values of *l* and *lx*, other parameters are fixed at: *w*_*1*_ = 0.5 mm, *w*_*2*_ = 0.2 mm, *g* = 0.2 mm, s = 2 mm and *ly* = 2.5 mm. (**a**) Transmission amplitudes. (**b**) Transmission phases.

**Figure 5:**
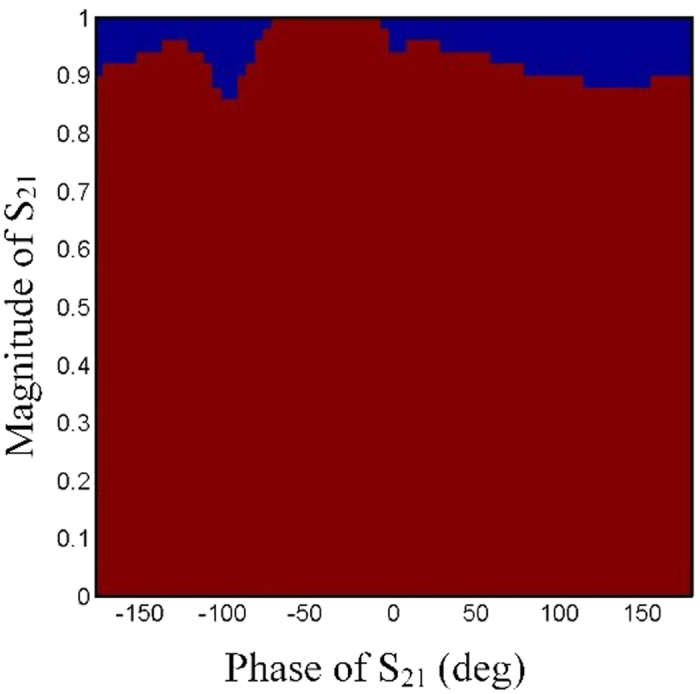
Value range of complex transmission coefficients. The red area shows the obtainable range, while the blue area shows the unobtainable range.

Figure 3 illustrates a subwavelength-scale Huygens particle, which is composed of two horizontal split-ring resonators (SRRs) and a vertical double-SRR, integrated on a dielectric substrate (*ε*_*r*_ = 2.65, tan *δ* = 0.009). The electric response is contributed by the horizontal SRRs in the middle layer, and the magnetic response is contributed by the vertical double-SRR. The independent controls of electric and magnetic responses are realized by adjusting the size of horizontal SRRs and vertical double-SRR. Full-wave simulations of the particle are executed in the commercial software, CST Microwave Studio (CST). In the procedures of designing unit cells, we found that magnetic responses mainly relate to *l* and electric responses mainly relate to *lx*, hence a database mapping transmission and reflection coefficients to the values of *l* and *lx* is established with the aids of CST. The other parameters are optimized by CST and finally are fixed as *w1 *= 0.5 mm, *w2 *= 0.2 mm, *g *= 0.2 mm, *s *= 2 mm and *ly* = 2.5 mm. When designing functional metasurfaces, we can search from the database for different sizes of the unit cells corresponding to different values of transmission and reflection coefficients. The transmission and reflection coefficients as functions of *l* (relating to magnetic responses) and *lx* (relating to electric responses) are given in Fig. 4, from which similar variation trends of amplitudes and phases are observed to those shown in Fig. 2. Figure 5 displays the available range in the Cartesian system. It is clearly demonstrated that arbitrary complex transmission coefficients with modulus values smaller than 0.85 is obtained. The transmission efficiency is much higher than that in previous works. In addition, the independent controls of transmission amplitudes and phases are not performed by rotating the particles, and hence little cross polarizations are introduced in the procedures.

## Designs and experiments

**Figure 6:**
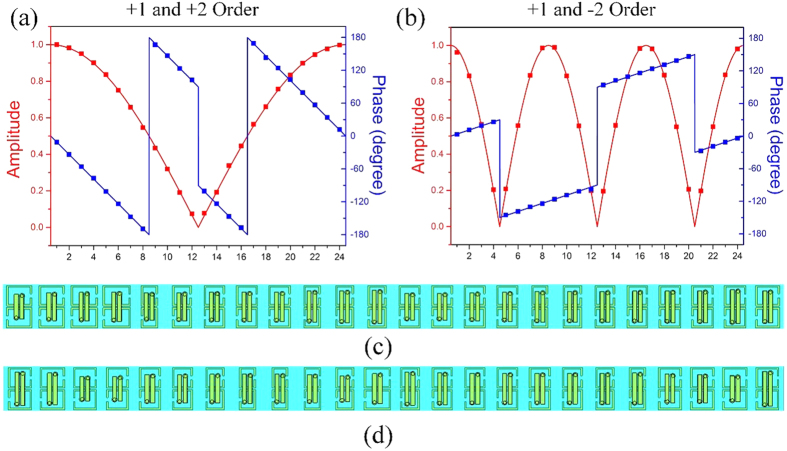
Amplitude and phase profiles of the metasurface gratings. Solid lines represent the calculated values, and symbols represent the designed values by using different particles. (**a**) Metasurface grating with *m* = +1 and *m* = +2; (**b**) Metasurface grating with *m* = +1 and *m* = −2; (**c**) One period of the simulation model of the metasurface grating with *m* = +1 and *m* = +2; (**d**) One period of the simulation model of the metasurface grating with *m* = +1 and *m* = −2.

**Figure 7:**
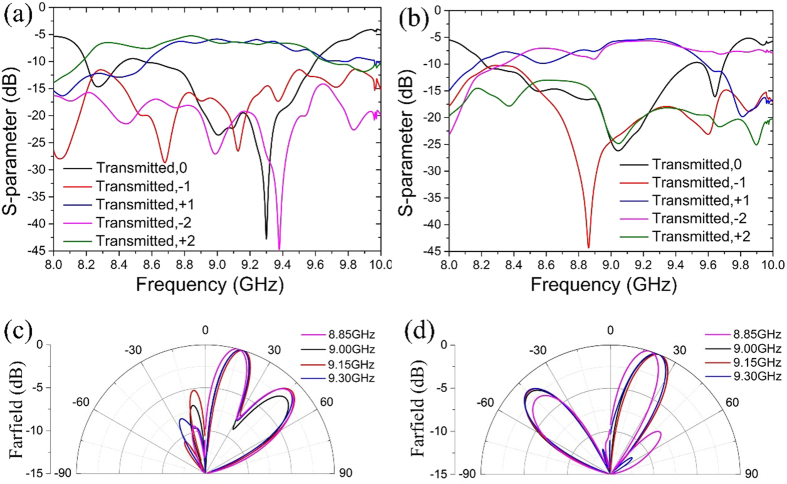
The simulated results of two metasurface gratings. (**a**) Floquet harmonics and (**c**) normalized farfield patterns of the metasurface grating with *m* = +1 and *m* = +2. (**b**) Floquet harmonics and (**d**) normalized farfield patterns of the metasurface grating with *m* = +1 and *m* = −2.

Two Huygens metasurface gratings that are able to produce multiple diffraction orders are designed using the Huygens particle. The general expression of transmission coefficient for the gratings are written as


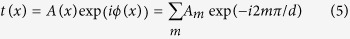


where *d* is the grating period and *m* is an integer denoting the diffraction order, *A*_*m*_ is amplitude of desired diffraction order. The first metasurface produce two diffraction orders corresponding to *m* = +1 and *m* = +2; the second metasurface produce two diffraction orders corresponding to *m* = +1 and *m* = −2. The amplitudes of all the diffraction orders are set to be identical. The period of the designed metasurface is 96 mm, and each period contains 24 particles. In Fig. 6, solid lines give the required profiles of transmission amplitudes and phases according to Eq. (5), while symbols show the designed values corresponding to different particles. Full-wave simulations are performed with the aids of commercial software (CST microwave studio). Floquet ports and unit-cell boundaries are applied to mimic the infinite metasurface gratings. Simulated Floquet harmonics in Fig. 7(a,b) verify that the desired diffraction orders are excited by the proposed metasurfaces at around 9 GHz. The normalized far-field patterns in Fig. 7(c,d) display the directions of the diffraction beams. The higher orders tends to away from the normal direction. The gain decreases of the higher-order diffraction beams is resulted from the fact that the scatted fields of Huygens particles are not uniform in free space.

**Figure 8:**
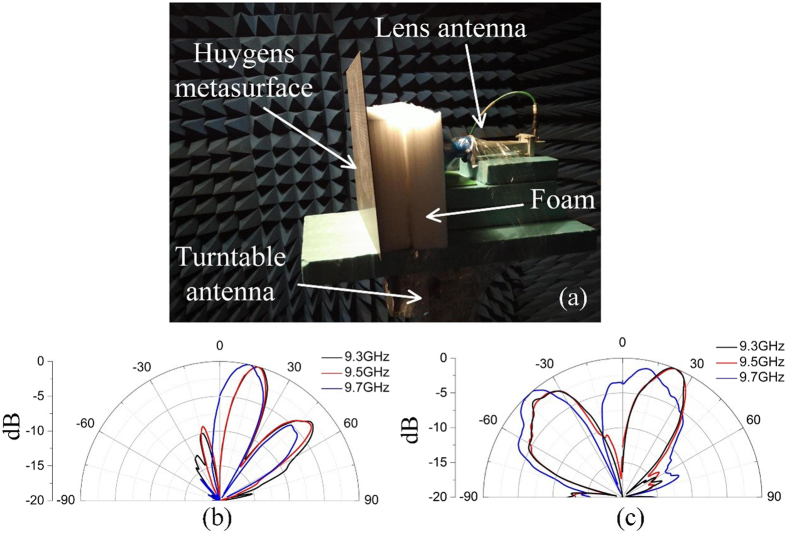
Measuring environment and measured far-field patterns. (**a**) The experimental platform. (**b**) The measured far-field patterns of the metasurface grating with *m* = +1 and *m* = +2. (**c**) The measured far-field patterns of the metasurface grating with *m* = +1 and *m* = −2.

The two designed metasurfaces are fabricated with the size of 288 mm × 297 mm. The experiments are taken in an anechoic chamber. The measuring environment is illustrated in Fig. 8(a). A metamaterial lens antenna^36^, which transforms the quasi-spherical waves into plane waves, serves as the source antenna in experiments. The diameter of antenna’s aperture is 100 mm. The lens antenna and the fabricated metasurface grating are separated by foams, and both of them are mounted on an antenna turntable. A standard horn antenna serves as the receiving antenna. The lens antenna and the standard horn are connected to two ports of an Agilent N5245A network analyzer. The measured far-field patterns of the diffraction beams are shown in Fig. 8(b) (for *m* = +1 and *m* = +2) and 8(c) (for *m* = +1 and *m* = −2). According to the measurements, the working frequencies are higher than those in simulations, and small deviations of the elevations angles are observed. These inconsistences mainly resulted from the reason that the incident plane waves are produced in near-field region by using metamaterials lens antenna. Multiple reflections between samples and source antenna destroy the uniformity of the plane waves. These problem can be alleviated by integrating the design of Huygens samples and source antennas. Nevertheless, reasonable agreements between simulations and measurements are observed.

## Conclusion

High-efficiency and independent controls of transmission amplitudes and phases are realized by using Huygens metasurfaces. Two metasurface gratings are combined with a metamaterials lens to control multiple diffractions beams. The proposed Huygens metasurfaces can be applied in synthesizing complex field patterns such as computer generated holography. The highly efficient and independent controls of transmission amplitudes and phases can also be used in designing low-sidelobe antennas. Compared with conventional metasurfaces, the proposed metasurface takes the advantages of arbitrarily designing multiple diffraction beams, hence can be used in multi-object monitorings and multi-channel communications.

## Additional Information

**How to cite this article**: Wan, X. *et al.* Independent modulations of the transmission amplitudes and phases by using Huygens metasurfaces. *Sci. Rep.* **6**, 25639; doi: 10.1038/srep25639 (2016).
